# HIV-1 drug resistance among people living with HIV receiving dolutegravir-based anti-retroviral regimens in Uganda: a national laboratory-based survey using remnant viral load samples, 2022

**DOI:** 10.1093/jac/dkaf180

**Published:** 2025-06-09

**Authors:** Christine Watera, Juliana de Fatima Da Silva, Grace Namayanja, Juliet Nkugwa Asio, Deogratius Ssemwanga, Sherri Pals, Miriam Nabukenya, Elliot Raizes, Maria Nanyonjo, Bill Elur, Esther Nazziwa, Grace Sanyu, Alisen Ayitewala, Mina Ssali, Cordelia Katureebe, Hudson Balidawa, Du-Ping Zheng, Clement Zeh, Stephanie Hackett, Christina Mwangi, Mary Naluguza, Jonathan Ntale, Edward Katongole Mbidde, Pontiano Kaleebu

**Affiliations:** Department of General Virololgy, Uganda Virus Research Institute (UVRI), Entebbe, Uganda; Division of Global HIV & TB, U.S. Centers for Disease Control and Prevention, Atlanta, USA; Division of Global HIV & TB, U.S. Centers for Disease Control and Prevention, Kampala, Uganda; Department of General Virololgy, Uganda Virus Research Institute (UVRI), Entebbe, Uganda; Department of General Virololgy, Uganda Virus Research Institute (UVRI), Entebbe, Uganda; Sequencing Platform, Medical Research Council (MRC)/UVRI & London School of Hygiene and Tropical Medicine (LSHTM) Uganda Research Unit, Entebbe, Uganda; Division of Global HIV & TB, U.S. Centers for Disease Control and Prevention, Atlanta, USA; Central Public Health Laboratories, Ministry of Health, Kampala, Uganda; Division of Global HIV & TB, U.S. Centers for Disease Control and Prevention, Atlanta, USA; Sequencing Platform, Medical Research Council (MRC)/UVRI & London School of Hygiene and Tropical Medicine (LSHTM) Uganda Research Unit, Entebbe, Uganda; Division of Global HIV & TB, U.S. Centers for Disease Control and Prevention, Kampala, Uganda; Division of Global HIV & TB, U.S. Centers for Disease Control and Prevention, Kampala, Uganda; Department of General Virololgy, Uganda Virus Research Institute (UVRI), Entebbe, Uganda; Central Public Health Laboratories, Ministry of Health, Kampala, Uganda; AIDS Control Program, Ministry of Health, Kampala, Uganda; AIDS Control Program, Ministry of Health, Kampala, Uganda; AIDS Control Program, Ministry of Health, Kampala, Uganda; Division of Global HIV & TB, U.S. Centers for Disease Control and Prevention, Atlanta, USA; Division of Global HIV & TB, U.S. Centers for Disease Control and Prevention, Atlanta, USA; Division of Global HIV & TB, U.S. Centers for Disease Control and Prevention, Atlanta, USA; Division of Global HIV & TB, U.S. Centers for Disease Control and Prevention, Kampala, Uganda; Division of Global HIV & TB, U.S. Centers for Disease Control and Prevention, Kampala, Uganda; Division of Global HIV & TB, U.S. Centers for Disease Control and Prevention, Kampala, Uganda; Department of General Virololgy, Uganda Virus Research Institute (UVRI), Entebbe, Uganda; Department of General Virololgy, Uganda Virus Research Institute (UVRI), Entebbe, Uganda; Sequencing Platform, Medical Research Council (MRC)/UVRI & London School of Hygiene and Tropical Medicine (LSHTM) Uganda Research Unit, Entebbe, Uganda

## Abstract

**Background and objectives:**

Uganda adopted dolutegravir as its preferred HIV treatment regimen in the national guidelines for treatment of HIV and AIDS in 2018. We conducted a survey to estimate dolutegravir resistance 4 years post-dolutegravir introduction in routine clinical settings. This was a cross-sectional survey to estimate the prevalence of HIV drug resistance (HIVDR) to dolutegravir among children and adults with viral non-suppression (VNS; ≥1000 copies/mL) receiving dolutegravir-based antiretroviral therapy for at least 9 months.

**Methods:**

We used remnant specimens from routine viral load monitoring stored at Central Public Health Laboratories during February–April 2022. Genotyping of the protease, reverse transcriptase and integrase regions of the HIV-1 pol gene was done using Thermo Fisher^®^ kits and analysed using the Stanford HIVDR database. Weighted prevalences of HIVDR with 95% confidence intervals (CI) were estimated for adults (≥15 years) and children (0–14 years).

**Results:**

We randomly selected 857 specimens including 457 from adults and 400 from children for HIVDR testing from 3578 eligible specimens collected during February–April 2022. Five hundred and eleven (59.6%) were successfully genotyped in the integrase region. Intermediate- to high-level dolutegravir HIVDR prevalence was 3.9% (CI: 0.7, 7.1) for adults and 6.6% (CI: 3.5, 9.6) for children.

**Conclusion:**

HIVDR to dolutegravir was uncommon but present among both children and adults with VNS after 9 months or more of exposure to dolutegravir. Additional longitudinal outcomes data are needed to determine if adherence counselling for patients with VNS on dolutegravir regimens might improve outcomes.

## Introduction

In a nationally representative survey among adults conducted in 2016, Uganda experienced high pre-treatment NNRTI resistance of 14.1%.^1^ In another survey among children less than 18 months recently diagnosed with HIV conducted in 2012, the prevalence of initial resistance to NNRTIs was 35.7%.^2^

Since 2018, WHO HIV treatment guidelines have recommended the combination of tenofovir disoproxil fumarate, lamivudine and dolutegravir (TLD) as the preferred first-line regimen for initiating ART among adults and adolescents living with HIV.^3^ In 2019, these guidelines were updated, and dolutegravir-based regimens (DBRs) were recommended by WHO as the preferred HIV treatment option in all populations.^4^ Dolutegravir is a potent antiretroviral drug with fewer side effects and high genetic barrier to resistance.^5^ In Uganda, the Ministry of Health (MoH) started a population-wide transition to TLD in 2018,^6^ and as of March 2023, >1.32 million people living with HIV (PLHIV) were receiving ART, of whom 1.28 million (97%) were on DBRs with an overall viral load suppression (VLS) rate of 94%.^7,8^

To monitor treatment effectiveness for PLHIV on ART, viral load (VL) testing in Uganda is conducted at 6 and 12 months of ART initiation and annually thereafter for adults, 6 monthly for children and adolescents and 3 monthly for pregnant and breast feeding women, with viral suppression, generally defined as VL results < 1000 copies/mL.^9^

Achieving high levels of VLS in populations taking ART prevents HIV transmission, associated morbidity and mortality and emergence of HIV drug resistance (HIVDR).^10^ However, there are limited data to guide empiric management of virological failure (VF) among patients receiving TLD, and therefore targeted patient level HIVDR testing in addition to routine VL monitoring and surveillance of emerging dolutegravir resistance is needed to avoid unnecessary switches and to inform national and global public health strategies.^11^ Surveillance provides the data that will drive policy. When we learn that a little over 3% of individuals with VF have HIVDR, we have solid grounds to create policy that discourages early switches upon viral non-suppression (VNS). Although resistance to dolutegravir is expected to be uncommon at the time of VF, it may emerge over time.^12^ In fact, emerging dolutegravir resistance has been reported from the Malawi HIV treatment program among people who spent a long time on failing ART^13^ and recently reported in US President’s Emergency Plan for AIDS Relief (PEPFAR) supported dolutegravir resistance surveys in Mozambique, Ukraine and Malawi.^14^ Emerging resistance in high-income settings where access to HIVDR testing is readily available was also recently reported.^15^

We utilized a laboratory-based methodology using remnant specimens from the national VL program to determine the prevalence of HIVDR among individuals receiving DBRs with VNS.

## Methods

### Study design and setting

VL testing for monitoring response to ART among PLHIV receiving care at approximately 2000 ART treatment centres in Uganda is performed in one national laboratory located at the Central Public Health Laboratory (CPHL) in Kampala. However, point of care VL testing is performed for special populations such as pregnant women and breastfeeding mothers at approximately 300 ART treatment centres.

We conducted a cross-sectional survey in partnership with U.S. Centers for Diseases Control and Prevention (CDC) to estimate the prevalence of HIVDR among PLHIVs receiving DBRs for at least 9 months with VNS (defined as any VL ≥ 1000 copies/mL) utilizing the cyclical acquired HIV drug resistance (CADRE) methodology that has previously been described.^16^

The survey utilized prospectively collected VL specimens from patients receiving DBRs. Remnant specimens from eligible dry blood spot (DBS) cards or plasma collected at the time of routine VL testing were retrieved from CPHL biorepository and shipped to the Medical Research Council (MRC)/Uganda Virus Research Institute (UVRI) and London School of Hygiene & Tropical Medicine (LSHTM) genotyping laboratory in Entebbe for HIVDR testing.

### Population and sampling

The survey specimens were selected from among eligible specimens tested for VL during February–April 2022 at CPHL. Specimens were obtained from PLHIV on DBRs for ≥9 months with VNS. Specimens were excluded if they had insufficient volume, were of poor quality or had no corresponding variables of interest within the VL laboratory information system (VL LIS). These variables were date of birth or age, sex and date of dolutegravir initiation.

Sample sizes of 457 for adults and 400 for children were estimated assuming confidence limits of ±3.3% for adults and ±3.1% for children, an HIVDR prevalence of 10%, a 70% PCR amplification rate and a finite population correction for children.

Specimens for adults were randomly selected in two equal proportions using simple random sampling from among those tested for VL and meeting all other eligibility criteria in February and March 2022, whereas specimens for children were selected in three equal proportions from among those tested in February, March and April 2022.

**Figure 1.**
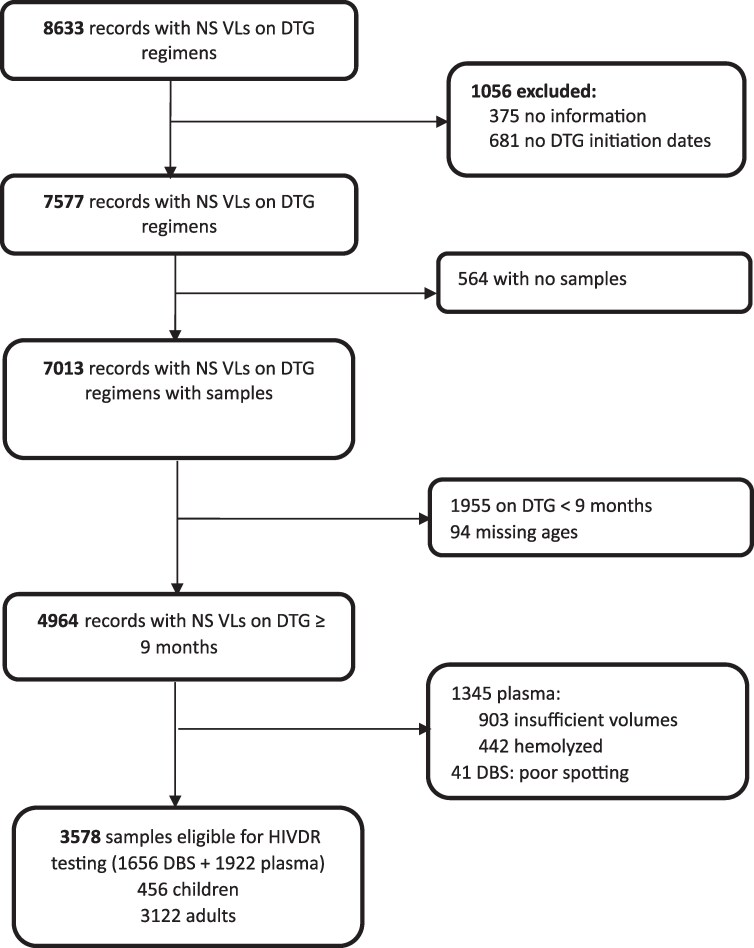
Study flow chart showing records and corresponding non-suppressed VL samples screened for DTG resistance survey in Uganda, 2022. DBS, dried blood spot; DTG, dolutegravir; HIVDR, HIV drug resistance; NS, non-suppressed; VL, viral load.

During the survey period, 398 663 specimens were tested for VL at CPHL, and among these, 300 307 (75.3%) were from PLHIV on DBRs. Among those on DBRs, 11 258 (3.7%) had VL ≥ 1000 copies/mL. After excluding all ineligible specimens, 3122 (87.3%) specimens for adults and 456 (12.7%) specimens for children were eligible for the survey (Figure 1).

Clinical and demographic data were abstracted from the VL LIS which contained data from laboratory requisition forms shipped with all specimens. Each specimen with its corresponding data was assigned a unique study identification number (SID).

### Laboratory procedures

#### Specimen retrieval

At CPHL, previously stored remnant DBS and plasma specimens with VNS results were retrieved from −80˚C storage and screened for suitability for genotyping. The specimen volume required was ≥500 µL of plasma or at least three DBS spots. The required specimen appearance was a well packaged, dry and properly saturated DBS card or properly processed plasma specimen with no indication of haemolysis. The specimen identification details were matched to corresponding details captured electronically prior to transfer into a cool box lined with dry ice. Batches of 10 individually packed DBS specimens were packaged in bigger zip-top bags while plasma specimens were placed in cryogenic sample boxes. The sealed cool boxes and specimen transportation logs were transferred to the MRC/UVRI and LSHTM Uganda Research Unit genotyping laboratory while maintaining the sample cold chain. Upon arrival in the genotyping laboratory, specimens were cross-checked to ensure that they were still frozen and immediately transferred to −80˚C storage prior to further laboratory processing.

#### Genotyping details

Genotypic resistance testing was done in the WHO-designated HIVDR laboratory at the MRC/UVRI and LSHTM Uganda Research Unit. Viral RNA was extracted from DBS (400 μL eluted from two spots) or 140 μL of plasma using the QIAGEN viral RNA extraction kit (QIAGEN, Hilden, Germany). Reverse transcription and nested PCR were done using the Thermo Fisher^®^ HIV-1 genotyping kit. The protease gene (1–99 amino acids), reverse transcriptase gene (1–252 amino acids) and the integrase gene (1–288 amino acids) were amplified. Cycle sequencing was done using the Thermo Fisher^®^ sequencing module in the kit. Sequencing was performed on the ABI 3500 genetic analyzer (Applied Biosystems). The sequence bases were checked/edited using a customized RECall software program version 2.7 from WHO^17^ and drug resistance mutations (DRMs) were analysed using the Stanford HIVdb Program using the 2022 WHO mutation list. HIV subtyping was performed using the REGA HIV-1 Subtyping Tool 3.0^18^ and the COMET-HIV-1 subtyping tools.^19^

For quality control purposes, our laboratory is enrolled in the Virology Quality Assurance Program and all sequences generated are assessed for cross-contamination by phylogenetic analysis using the molecular evolutionary genetics analysis tool (MEGA 7.0). Further quality assurance using the WHO/British Columbia Center for Excellence in HIV/AIDS HIV drug resistance quality control tool was done to get good quality sequences free from contamination, stop codons and APOBEC mutations.

### Statistical analysis

#### Data management and analysis

The abstracted participant’s clinical and demographic data were linked to the laboratory data consisting of VL and HIVDR results using the SID to create an analysis data set.

HIVDR to dolutegravir was defined as the presence of HIV DRMs in the sequences classified as low-, intermediate- or high-level resistance by the Stanford HIVdb. Any HIVDR was considered as sequences containing at least one DRM to any of these drug classes: NNRTI, NRTI, PI or integrase strand transfer inhibitors (INSTIs). All statistical analyses were performed using SAS version 9.4. Prevalence of HIVDR with 95% CIs was estimated for adults and children separately.

For the estimates of HIVDR prevalence and 95% confidence limits, we incorporated two types of weights: first, design weights to account for the differential probability of specimen selection by month and second, weights to account for the missingness due to failure of specimens to amplify. For the second category of weights, we fitted a propensity score model predicting amplification and including VL category, specimen type, the interaction of VL category and specimen type, age, sex and region as covariates and weighted within propensity score classes.

#### Ethical considerations

This activity was reviewed by the UVRI Research Ethics Committee (GC/127/834) and the Uganda National Council for Science and Technology (HS 1774 ES), as well as CDC, was deemed not to be research and was conducted consistent with applicable federal law and CDC policy: 45 C.F.R. part 46.102(l)(2); 21 C.F.R. part 56; 42 U.S.C. §241(d); 5 U.S.C. §552a; and 44 U.S.C. §3501 et seq. No contact with participants occurred during this assessment, and secondary data collection was done using patient records.

## Results

### Participants characteristics

During February–April 2022, 857 (24.0%) out of the 3578 eligible remnant blood specimens from the national VL testing program (Figure 1) were sampled and included in the survey. Of these, 457 (53.3%) were from adults and 400 (46.7%) from children. The reason for VL testing was routine client monitoring for 92.2% of the participants whose specimens were included in the survey and repeat VL testing after intensive adherence counselling (IAC) for 5.4%.

The median age of the adults was 34 [interquartile range (IQR): 26–43] years and 11 (IQR: 10–13) years for children. Two hundred and eighty-four (62.1%) of adults and 211 (52.8%) of children were female. Median duration on DBRs was 18 (IQR: 13–27) months for adults and 15.5 (IQR: 11–22) months for children (Table 1).

**Table 1.** CADRE_UGANDA participant characteristics, 2022

Characteristic	Children (0–14 years)		Adults 15+ years	
	All samples (*n* = 400)	Amplified samples (*n* = 256)^a^	All samples (*n* = 457)	Amplified samples (*n* = 255)^a^
Sex				
Female	211 (52.8)	129 (50.4)	284 (62.1)	159 (62.4)
Male	189 (47.3)	127 (49.6)	173 (37.9)	96 (37.7)
Median age in years (IQR)	11 (10–13)	11 (10–13)	34 (26–43)	33 (25–44)
Median duration on all ART (including the time on DTG) in months	84.7 (45.1–108.8)	85.8 (46.8–110.2)	59 (28.3–96.0)	55.1 (24.0–102.5)
Median duration on DTG-based regimen	14.9 (10.6–22.0)	15.9 (10.7–21.6)	18 (13–27)	17.1 (11.9–25.2)
Central (Central 1 and 2, Kampala)	101 (25.3)	71 (27.7)	135 (29.5)	73 (28.6)
East (East Central, Mid-Eastern)	67 (16.8)	42 (16.4)	71 (15.5)	35 (13.7)
West (South-Western, Mid-Western)	71 (17.8)	35 (13.7)	123 (26.9)	55 (21.6)
North (Mid-North, North-East, West Nile)	161 (40.3)	108 (42.2)	128 (28.0)	92 (36.1)
Sample type				
DBS	162 (40.5)	50 (19.5)	227 (49.7)	74 (29.0)
Plasma	238 (59.5)	206 (80.5)	230 (50.3)	181 (71.0)
Reason for VL testing				
Routine	368 (92.0)		423 (92.6)	
Repeat	28 (6.2)		19 (4.2)	
Antenatal care	1 (0.3)		12 (2.6)	
Missing	3 (0.8)		3 (0.7)	
Amplification success rate (%)—INSTI				
DBS	50/162 (30.9)	—	74/227 (32.6)	—
Plasma	206/238 (86.6)	—	181/230 (78.7)	—
VL category				
1000–3999	127 (31.8)	58 (22.7)	186 (40.7)	77 (30.2)
4000–9999	85 (21.3)	61 (23.8)	73 (16.0)	38 (14.9)
10 000–99 999	130 (32.5)	89 (34.8)	133 (29.1)	89 (34.9)
≥100 000	58 (14.5)	48 (18.8)	65 (14.2)	51 (20.0)
HIV-1 subtype *N* (%) in pol region				
A	—	108 (56.3)	—	95 (51.1)
D	—	37 (19.3)	—	35 (18.8)
Recombinants	—	36 (18.8)	—	45 (24.2)
Other subtypes	—	2 (1.0)	—	4 (2.2)
Unique recombinant forms	—	9 (4.7)	—	7 (3.8)

DTG, dolutegravir.

^a^Amplification in integrase region.

### Amplification rates by sample type and VL category

Five hundred and eleven (59.6%) of the 857 specimens were successfully genotyped in the integrase region and passed the quality-control checks. Of these, 255 (55.7%) were from adults and 256 (64.0%) from children. Amplification was higher for plasma compared to DBS specimens [plasma = 83% (387/468); DBS = 31.8% (124/389)]. Amplification was >80% for plasma specimens with VL copies ≥ 10 000 copies/mL for both PR/RT and integrase regions (Table 2).

**Table 2.** CADRE_UGANDA amplification rates by sample type and viral load, 2022

	No. of specimens *N*	Integrase region of pol gene *n* (%)	PR/RT region of pol gene *n* (%)
Viral load, copies/mL		Plasma	
1000–3999	136	107 (78.7)	103 (75.7)
4000–9999	88	72 (81.8)	64 (72.7)
10 000–99 999	154	128 (83.1)	130 (84.4)
>100 000	90	80 (88.9)	76 (84.4)
Viral load, copies/mL		DBS	
1000–3999	177	28 (15.8)	15 (8.5)
4000–9999	70	27 (38.6)	17 (24.3)
10 000–99 999	109	50 (45.9)	42 (38.5)
>100 000	33	19 (57.6)	14 (42.4)

HIV-1 subtyping was performed in the integrase and the PR-RT regions and was successful for 378 specimens in both regions. HIV-1 subtypes observed were predominantly A (203; 53.7%) followed by recombinants (81; 21.4%) and D (72; 19.0%). The rest were other subtypes and unique recombinant forms. Subtypes by age category are shown in Table 1.

### Prevalence of resistance to integrase strand transfer inhibitors

Thirty-three (6.4%) of the 511 participants had major INSTI resistance mutations. Thirty-one (93.9%) of these participants had mutations conferring resistance to dolutegravir. Twelve (36.4%) of the 33 participants also had accessory INSTI mutations, mainly T97A (4) and E157Q (4). Overall, 74 (14.5%) of the 511 participants had accessory mutations.

The weighted prevalence of major INSTI resistance mutations was 4.4 (95% CI: 1.1, 7.6) and 7.6 (95% CI: 4.4, 10.9) for adults and children respectively.

**Figure 2.**
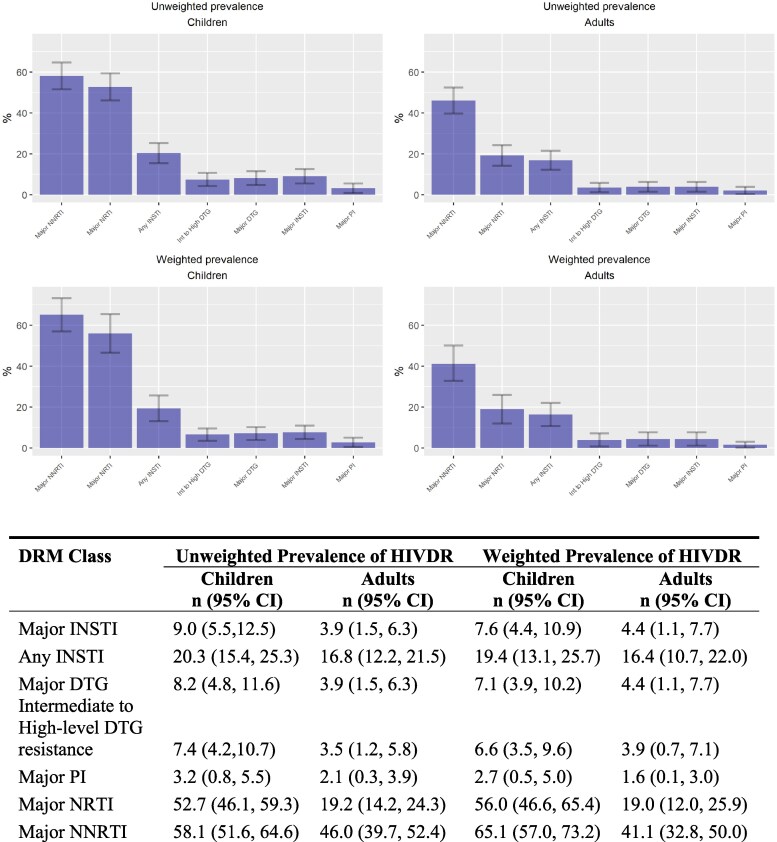
CADRE_UGANDA Prevalence of HIV DRM by DRM Class, 2022.

INSTI, integrase strand transfer inhibitor; DTG, dolutegravir; DRM, drug resistance mutation; NRTI, nucleoside reverse transcriptase inhibitor; NNRTI, non-nucleoside reverse transcriptase inhibitor; PI, protease inhibitor.

The weighted prevalence of major dolutegravir HIVDR mutations was 4.4% (95% CI: 1.1, 7.7) for adults and 7.1% (95% CI: 3.9, 10.2) for children. The prevalence of intermediate and high level dolutegravir resistance was 3.9% (95% CI: 0.7, 7.1) for adults and 6.6% (95% CI: 3.5, 9.6) for children. We had no samples with low-level resistance (Figure 2).

Among participants with major INSTI mutations, 17 (51.5%) had NRTI mutations, 16 (48.5%) had NNRTI mutations and 1 (3.0%) had PI mutations.

### Pattern of INSTI DRMs among adults and children receiving dolutegravir-based ART regimens

**Figure 3.**
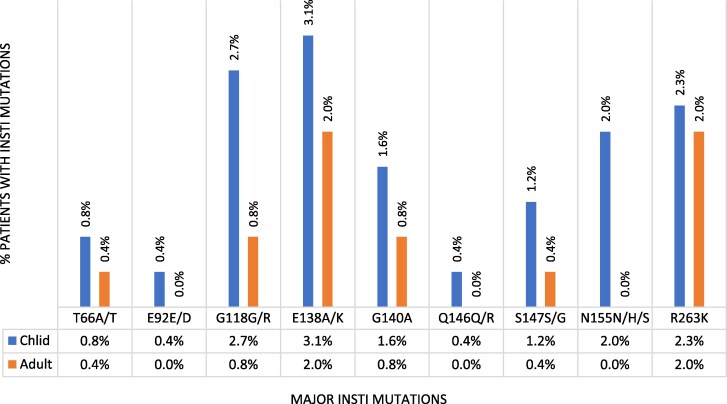
Patterns of major INSTI mutations among CADRE study participants in Uganda, 2022.

The most frequently observed major INSTI mutations were E138K/A in 13 (2.5%) including 8 (3.1%) children and 5 (2.0%) adults followed by R263K in 11 (2.2%) participants, including 6 (2.3%) children and 5 (2.0%) adults. Others were G118R/G in nine (1.8%), Q148K/R/Q in six (1.2%), G140A/G in six (1.2%) and N155H/N/S in five (1.0%) participants. All mutations were more frequently observed among children compared to adults (Figure 3).

Accessory mutations most frequently observed were T97A (in 45 participants), L74M/L (in 12 participants), S153A (in 8 participants) and E157Q (in 7 participants). Less commonly observed were H51Y, A128A, Q95K, G140GE and G163GR.

### Prevalence of resistance to PIs, NRTIs and NNRTIs

Of the 857 study specimens, 461(53.8%) were successfully genotyped in the PR/RT regions of the pol gene. Overall, the number of specimens with NRTI, NNRTI and PI mutations was 162 (35.1%), 238 (51.6%) and 12 (2.6%), respectively. The weighted prevalence of NRTI mutations was 19.0% (95% CI: 12.0, 25.9) among adults and 56.0% (95% CI: 46.6, 65.4) among children; the prevalence of NNRTI mutations was 41.1% (95% CI: 32.8, 50.0) among adults and 65.1% (95% CI: 57.0, 73.2) among children and that of PI mutations was 1.6% (95% CI: 0.1, 3.0) among adults and 2.7% (95% CI: 0.5, 5.0) among children (Figure 2).

## Discussion

Although the prevalence of dolutegravir resistance was low in this national laboratory-based survey for HIVDR among patients with VNS, it is higher than what was reported in clinical trials. In our study, the prevalence of major dolutegravir mutations was 4.4% among adults and 7.1% among children. These findings were similar to what was reported in a multi-cohort analysis^15^ and in Tanzania.^20^ Similar studies supported by PEPFAR investigating emerging drug resistance to dolutegravir in Malawi and Mozambique^14^ found higher dolutegravir resistance among adults, at 8.6% and 19.7%, respectively. This may be partially explained by the fact that in these cohorts, completion of adherence counselling was an inclusion criterion, in contrast, less than 10% of specimens in our study included specimens from individuals who had completed adherence counselling. Secondly, unlike other countries that transitioned all PLHIVs regardless of suppression status, Uganda transitioned individuals on first-line non-DBRs who were virally suppressed to DBRs and also offered TLD as second-line for individuals with known VNS.

The most common mutations we observed were E138K/A, followed by R263K. Others were G118R/G, Q148K/R/Q, G140A/G and N155H/N/S. Most of these mutations have been observed in other populations in sub-Saharan Africa and elsewhere.^20–23^ Accessory mutations seen in our study were also not very different from what had been reported earlier in Uganda.^24,25^ T97A has been reported to be present in between 5% and 10% of INSTI naïve individuals infected with subtype A virus,^26^ which is the commonest subtype in Uganda. T97A is associated with high-level resistance to dolutegravir when in the presence of major INSTI mutations.^27^ Though baseline resistance was not performed prior to ART initiation nor before switching clients to DBRs, it is unlikely that some of these individuals had been exposed to INSTIs since these drugs were not in common use.

Approximately half of the specimens with major INSTI mutations also had NRTI mutations and NNRTI mutations. It is possible that although these individuals were transitioned when virally suppressed, there were pre-existing resistance mutations to NRTI and NNRTI that had not yet reverted to wild-type virus. Some studies have shown a relationship between NRTI and NNRTI and dolutegravir resistance development.^15,28^

The genotypes we observed in the integrase region are reflective of the general HIV subtype prevalence in our population with a dominance of subtype A and unique recombinants.^29^ However, the numbers were too small to relate HIVDR with subtypes.

There are very few data reported for dolutegravir resistance among children; the higher prevalence in children < 15 years, although not statistically significant, was not surprising due to the adherence challenges children generally face leading to higher VNS.^30,31^

Studies have shown that the majority of individuals with VNS while on DBRs do suppress after IAC, suggesting that adherence remains the main challenge especially in children.^32^ However, this analysis was not done in our study because of its cross-sectional design. In order to preserve the effectiveness of dolutegravir, adherence support through ART programs might need to be improved. This might include scaling up VL monitoring to quickly identify patients that need IAC. Given that the proportion of PLHIV with DRMs to dolutegravir among those with VNS is low, improving adherence among both children and adults in Uganda might be beneficial.^33,34^ There are efforts to provide multi-month ART refills, treatment and VL literacy for adults and adolescents so that they are educated to know the benefits of ART and meaning of VL results.^35^ The youth and adolescent peer supporters track peers for HIV testing and support their linkage to care, provide adherence counselling and track those who miss appointments. In addition, PEPFAR supports the integrated service delivery model that reaches out to non-suppressed PLHIV who are not able to come to health facilities with various services including ART refills, adherence counselling and directly observed treatment support.^35–37^

To strengthen adherence in children, PEPFAR Uganda in collaboration with MoH supports caregiver treatment literacy, linkage of children and adolescents living with HIV to orphans and vulnerable children services for psychosocial support and community health caregiver directly observed treatment support. Finally, as the cost and complexity of routine genotyping remains high, there is a need for decision making tools that better triage individuals with the highest risk of HIVDR to undergo genotyping; these algorithms may take into consideration risk factors such as NRTI resistance, as well as rapid tests that help identify those with adherence barriers, such as urine tenofovir assays.^38,39^

With the efforts being made to introduce INSTI-based regimens in prevention as pre-exposure prophylaxis through the use of long-acting cabotegravir, it is crucial to preserve this drug class effectiveness, by continuous drug resistance surveillance. Accordingly, we plan to continue annual cycles of drug resistance monitoring using the CDC laboratory-based approach.

Our study has a major strength of utilizing specimens from all regions of the country cutting across different geographical regions, health facility level and specialization, ownership of the ART clinic and other categories. This makes the results that we have observed nationally representative by reducing the selection bias associated with utilizing samples from a limited number of clinics out of the many in Uganda. In addition, the design of using specimens for genotyping from a central repository makes the implementation of the study logistically easier and also cheaper with results available in a short time to enable programmatic decisions for the national ART program.

There were some limitations in our study. The genotype success rate was 59.6% with the lowest success attributed to the DBS specimens. We have previously reported the challenges of genotyping DBS, particularly specimens obtained for routine ART program client management.^40^ To attempt to mitigate the bias introduced by the high percentage of specimens that failed to amplify, we fitted a propensity score model predicting amplification that included specimen type, VL category and other variables and then weighted within propensity score category. While this approach could yield an improved HIVDR estimate when the variables included in the model are strongly related to both missingness and the prevalence of drug resistance, but still must be interpreted cautiously because of the high percentage of missingness. Secondly, because of the cross-sectional approach of the study, we were unable to ascertain whether the mutations observed have implications on VL suppression and also distinguish between pre-treatment (transmitted raltegravir associated DRMs or polymorphisms) and acquired mutations/drug resistance. The clinical significance of DRMs to dolutegravir remains an important research gap. Previous data show that the presence of NRTI resistance does not adversely affect clinical outcomes as predicted by genotype,^18^ but further research is needed to assess if this also applies to INSTIs. This would have important public health implications, as currently patients failing a DBR with drug resistance to dolutegravir are commonly managed with a switch to a darunavir-based regimen or an increase in dolutegravir dose, both of which are more expensive and have a higher pill count, further burdening those who struggle with adherence.

Furthermore, some studies have postulated the presence of NRTI or NNRTI resistance to be a risk factor to drug resistance to dolutegravir, but we did not observe enough dolutegravir resistance events to analyse the association of factors in this study such as presence of NRTI/NNRTI mutations, sex and subtype with dolutegravir resistance.

### Conclusion

Resistance to dolutegravir was uncommon among both children and adults with VNS after 9 months or more of exposure to dolutegravir. We did not observe resistance mutations to dolutegravir in most of the participants, and our results suggest that dolutegravir remains effective and that most individuals with VNS will probably not benefit from a regimen change but probably from strengthened adherence support. Ongoing HIV drug resistance surveillance and expanded adherence support might be helpful in preventing acquired HIVDR among individuals with VNS on dolutegravir-containing regimens.
